# A concept mobility device with multi-positional configurations and child-kind restraint for safe perioperative transfer and induction of anaesthesia in children with autistic spectrum disorder – a cross sectional study

**DOI:** 10.1186/s12913-021-06309-3

**Published:** 2021-04-09

**Authors:** Hwan Ing Hee, Ying Ching Lim, Tracy Tan, Sharon Wan, Olivia Wijeweera, Sumin Lee, Kavitha Raghavan

**Affiliations:** 1 Department of Paediatric Anaesthesia, KK Women’s and Children’s Hospital; Anaesthesiology and Peri-operative Science Duke–NUS Medical School, 100 Bukit Timah Road, Singapore, 229899 Singapore; 2 Department of Anaesthesia, Khoo Teck Puat Hospital, Singapore, Singapore; 3 Paediatric Anaesthesiology, St. Jude Children’s Research Hospital, Memphis, USA

**Keywords:** Autistic spectrum disorder, Perioperative movement, Perioperative transport, Hospital mobility device, Induction of Anaesthesia

## Abstract

**Background:**

Transfer into the operating room, onto the operating table and mask induction of anaesthesia are major challenges faced by children with Autistic Spectrum Disorder (ASD). In a pilot study, parents observed that perioperative transfer becomes unsafe and difficult when children with ASD becomes uncooperative.

**Method:**

A CHILD-KIND CONCEPT mobility system comprising of multi-positioning seat configurations and restraining module was developed with inputs from multi-disciplinary healthcare professionals and parents with children with ASD. To appeal to children and motivate child-machine interaction, the seat configurations and restraining module are designed to take the form of child-friendly, non-threatening, fun and familiar items. The sitting configuration, sitting to supine transformation, the restraint modules resemble racing-car seat, reclining motion of a home massage chair, safety restraints found in airplanes and amusement rides respectively. Healthcare professionals (HCPs) involved in the perioperative patient care, parents of ASD children and children (neurotypical and ASD) experience the use of the system in a non-clinical environment and participated in a survey study. The acceptance of its functionality (HCPs, parents) for perioperative transfer and induction of anaesthesia, rating of the user experience and likes and dislikes of (parents and children) were obtained.

**Results:**

Thirty-two HCPs, 30 parents and 23 children participated. Majority of parents and HCPs opined the use of the system enables improvement in the management of perioperative movement (90.0% parents, 100% HCPs), safe perioperative movement (86.7% parents, 96.9% HCPs) and promotes ease of anaesthesia induction (76.7% parents, 90.6% HCPs) for uncooperative combative ASD children. Overall, 93.8% HCPs and 86.7% parents would recommend its frequent use in their own practice and their ASD children respectively. Attractiveness and multi-functionality are attributes endorsed by parents and children. Children endorse its use for induction of anaesthesia (73.9%), dental chair (82.6%), intra-hospital transfer (95.7%).

**Conclusion:**

A child-kind mobility device that integrates appeal with functionality of restraint and multi-positional transformation has a potential to promote safe perioperative movement and ease of induction of anaesthesia in anxious uncooperative ASD children.

## Introduction

Autistic Spectrum Disorder (ASD) is a heterogenous neurodevelopmental disorder [1, 2] characterized by deficiencies in social skills, language development, restrictive interest and inability to cope with a change from routine. Children with ASD can also have anxiety disorders and sensory hypersensitivities [3, 4]. Many also have impairment in motor development [4–6], these mobility needs are often unrecognized [7].

Perioperative hospital visit is a stressful experience for these children for several reasons. Operating theatre (OT) environment presents multiple sensory stimuli such as unfamiliar crowd, lights, noises for children with sensory hypersensitivities. Routine activities embedded in admission processes such as waiting, clinical parameter measurements and patient transfers to the OT and operating room (OR) adds further stress. Movement onto the OT table and placement of face mask during induction of anaesthesia are have been identified as perioperative challenges [8]. When children are overtly anxious and agitated, they respond with a meltdown behaviour that may manifest as ‘flight’, ‘fight’ or even self-harm.

There is growing recognition within community, schools, hospitals [3] to support the needs of children with ASD. In recent years, policy statements have been established to promote road safety for safe transportation of children with special needs [9, 10]. However, reports relating to perioperative transfer and safety within hospital is limited. While roller and transfer boards are assistive equipment widely used for patient transfer [11], guidelines from Association of periOperative Registered Nurses (AORN) on safe patient handling and movement had recommended using safe patient handling equipment technology in the perioperative setting for moving and transferring patient that is based on patient’s unique handling and movement needs [11–13]. In our pilot study, parents of ASD children observed that perioperative transfer becomes unsafe and difficult when children become uncooperative. We developed a concept specialized child-kind mobility device in an attempt to meet the mobility needs of ASD children [14, 15]. The goal is to enhance safe and efficient process in (a) moving child to the OR (b) facilitate induction of anaesthesia and (c) transfer of anaesthetised children onto the operating table. We hypothesize that a child-kind approach by appealing to child’s perception of fun and familiarity will motivate better child-machine interaction, thereby facilitating the performance of the function of the mobility device.

The primary aim of this study is to evaluate healthcare providers' (HCPs) and parents’ opinion on the potential functionality of the proposed mobility system for safe restraint, induction of anaesthesia and transfer of children in the OR. The secondary aim is to evaluate the general experience of such a system by parents and children.

## Methodology

This is a prospective cross-sectional observation and survey study conducted with institutional ethics review board approval (SingHealth CIRB 2017/3127) and informed consent between April 2018 to November 2019. The review included the study protocol and took into consideration measures to mitigate fall risk and the use of seat belts as ‘restraint’ in the mobility system. The study complies with the standards, guidelines and regulations for clinical research.

### Specialized design mobility equipment

**Fig. 1:**
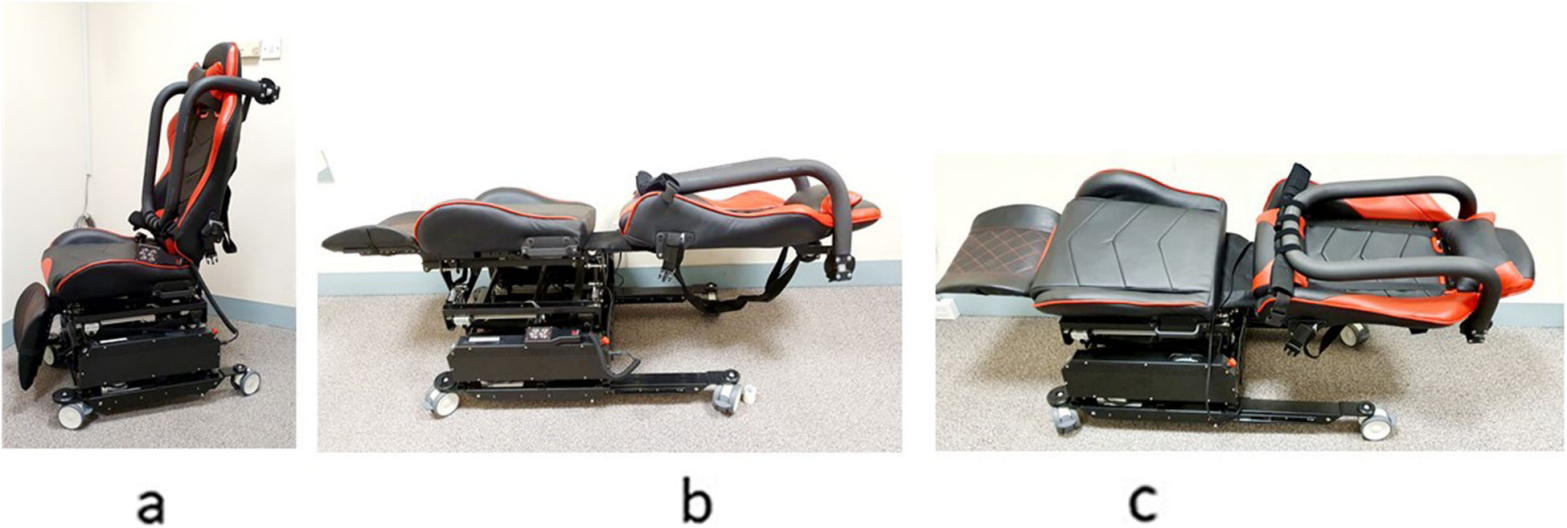
Illustration of the Specialized Apparatus for Transport, I-MOVE in (**a**) sitting position (**b**) supine position (**c**) lateral tilt

A specialized mobility system (I-MOVE) is developed as a CONCEPT CHILD-KIND MOBILITY DEVICE to interest children and initiate child-machine interaction that includes sitting on it in the ‘chair mode’, self-application of the restraint module and assisted ambulation [15]. See Fig. 1. This was developed as a collaboration between investigators from the paediatric anaesthesia department and biomedical engineers with input from parents with ASD children as well as multi-disciplinary healthcare professionals with experience in the care of children with ASD [15]. The mobility system is a multi-position configuration mobile chair-bed system with a restraining module [15, 16]. These positions are ‘sitting’ for preoperative sedation and induction of anaesthesia, ‘supine’ for airway management and transfer, ‘lateral tilt’ to aid lateral transfer onto the OT table, ‘Trendelenburg’ for oropharyngeal suctioning as part of airway protection [15]. These movements were activated via hand-held remote. In the resting position, it resembles a racing car chair, transformation to supine position resembles a reclining massage chair, all of which are different forms of chairs children are familiar with.

The restraint module is a key feature and comes in 3 options [15]; an over the hip adjustable restraint (like an airplane seat belt), an overhead restraint (‘amusement park restraint-like’) or a combination of the two with the strap traversing through the overhead restraint. The amusement park design adds to the contextual fun-based approach to I-MOVE. This reduces manpower and physical restraint required during patient transfer into the OR as well as patient handling, lifting and transfer during perioperative patient care.

### Survey participants and method

Survey participants included healthcare professionals (HCP), parents and children. The inclusion criteria are HCPs with experience in the perioperative care of ASD children in the operating theatre, these include operating theatre scrub and anaesthesia nurses, anaesthetists, surgeons and operating theatre technician with experience in the transfer and lifting of ASD children. The inclusion criteria for parents are those with children diagnosed with ASD. Permanent staff of paediatric anaesthesia department were excluded from the study to avoid conflict of interest.

The inclusion criteria for child participants were neurotypical children or ASD children aged between 6 to 16 years old and whom parents have confidence in their ability to complete the survey independently. Children with uncontrolled disruptive behaviour were excluded.

Informed consent was obtained from HCPs and parents. Parental consent was obtained for children with further assent obtained from children as deemed appropriate.

### Procedure

Parents and children were introduced to the operating theatre waiting area and the hospital trolley. The rest of the study was performed in a dedicated hospital room where I-MOVE was located and participants were informed about the motivation for development of the I-MOVE. The stage of Proof of concept of I-MOVE was emphasized. For the children, I-MOVE was introduced as a ‘special chair concept’ to help children move around in the hospital and to ‘go to sleep’. This was followed by a demonstration of the operation and function of I-MOVE in all configurations. HCPs and parents were instructed to examine and operate the system. Children were invited to explore the I-move such as sit on it and putting on the restraint module. This was followed by a survey.

### Questionnaire measures

In the survey, HCPs and parents answered eight closed ended 7-points Likert questions which evaluate their acceptance of multiple functions of I-move on anxious and uncooperative ASD children based on their work experience (HCPs) and personal experience (parents). Parents and children rated their general experience of the special chair using 5 points Likert scale and explained their likes and dislikes. HCPs further answered another 10 closed ended 5-points Likert questions on the usability of the I-move system based on System Usability Scale (SUS). Parents were asked on their opinion on the suitability of use of I-move and restraint in various situations. Children indicated the situations in which they would use the I-move.

### Statistics

Frequency was presented as numbers and percentage. For data analysis, methods used were t test for continuous data and chi square test for dichotomous data using SPSS version 19 (Inc Chicago IL, US). A *p* value of less than 0.05 was considered statistical significance.

In a previous observation pilot survey, we found that 25% of our parents would use the traditional hospital mobility device such as hospital trolley and wheelchairs. Parental opinion was used in the sample size calculation as parents of ASD children are the best resource to create a successful experience for their children [8]. We hypothesize that if the specially designed mobility device could garner a 75% agreement for use as a perioperative special mobility device, at a power of 0.9, 23 participants were needed. Further, if positive feedback on utility is taken as range from more or less agreed to strongly agreed, for a precision of 10% in the proportion of participants who give positive feedback on functional utility, assuming that the true proportion is 90%, a sample size of 27 is required for each expert group.

## Results

Thirty-two healthcare professionals, 30 parents of ASD children and 23 children took part in the study. Of the 23 children, 14 had ASD. The demographic baseline characteristics is summarized in Table 1. There is significant difference in gender and racial distribution between HCPs and parents (*p* < 0.001). HCPs were predominantly female and multi-racial. There is no statistical significance between neurotypical and ASD children with regards to the baseline characteristics.

**Table 1:** Baseline characteristics of participants

	HCPs *N* = 32	Parents *N* = 30	
Age in years, mean (SD)	39.66(10.62)	43.9(4.73)	
Gender, N (%) Male: Female	3(9.38): 29(90.63)	10(33.3):20(66.7)	
Race, N (%) Chinese: Malay: Indian: Others	18:1:0:13	23:1:6:0	
Years of work experience, mean (SD)	13.64(9.79)	NA	
Job scope, N (%): Nurse: Doctor: OTA	25(78.13):6(18.8):1(3.12)	NA	
GA experience, N (%)	32 (100.0)	13(43.3)	
	**Neurotypical** **Children** ***N*** **= 9**	**ASD** **Children** ***N*** **= 14**	**All Children** ***N*** **= 23**
Age in years, mean (SD)	10.61(3.28)	9.82(1.44)	10.12(2.30)
Gender, N (%) Male: Female	4(44.4): 5(55.6)	11(78.6): 3(21.4)	15(65.22): 8(34.78)
History of previous GA experience, N (%)	2(25.0)	8(57.1)	19:0:4:0
Years since last GA, mean (SD)	6 (1.41)	6.25(3.01)	6.20(2.70)
Weight in kg, mean (SD)	37.11(16.63)	37.06(11.08)	37.08(2.70)
Height in kg, mean (SD)	144.89(15.84)	141.89(9.84)	143.07(12.28)

*GA* general anaesthesia, *OTTA* operating theatre assistant

### Functionality of I-MOVE

**Fig. 2:**
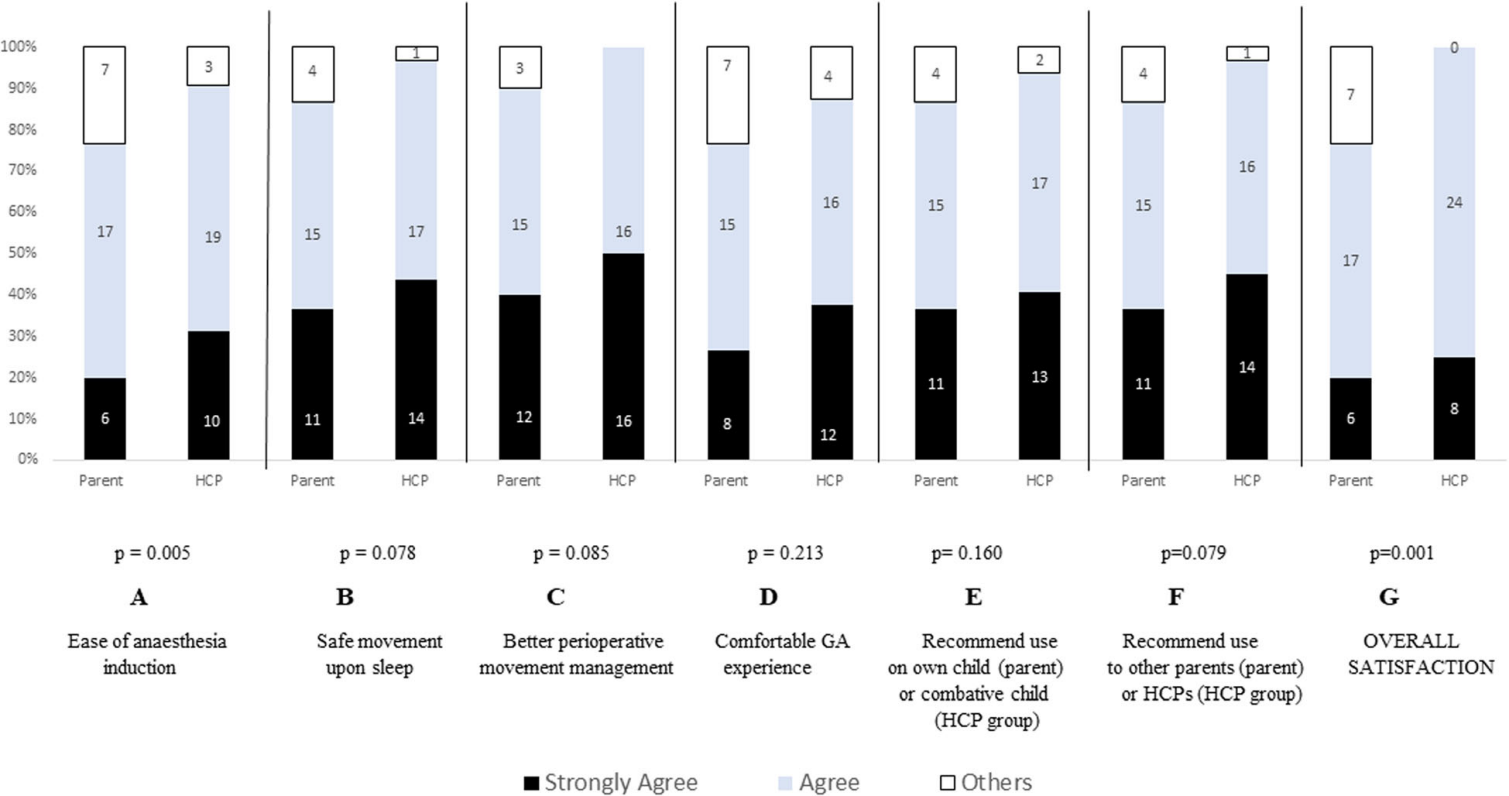
Comparison between HCPs' and parents' rating of the I-MOVE functionality. GA; general anaesthesia. Others include rating of More or less agree, undecided, more or less disagree, disagree and strongly disagree

Parental and HCPs perceptions on the functionality of I-move is summarized in Fig. 2. Twenty-nine parents (96.7%) opined that such a system would lead to a better management of combative children (12 Strongly agree, 13 agree, 4 more or less agree). All HCPs found the menu control for operations of the multi-positional transformation easy to use.

### General acceptance of I-MOVE

**Fig. 3:**
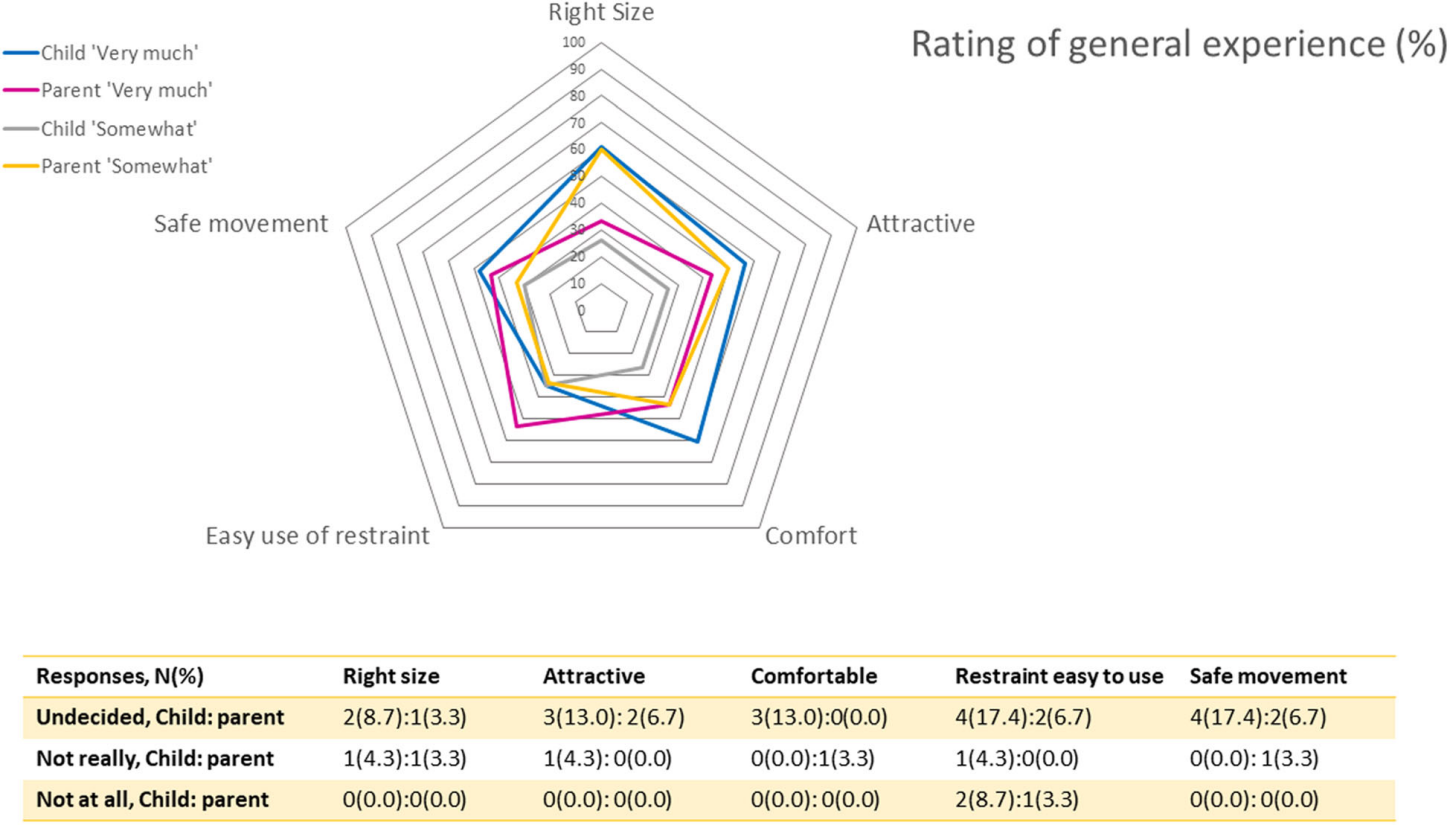
Comparison between parents and children on overall experience of I-MOVE

Comparison of parental and Child’s opinion on general experience is shown in Fig. 3. The size, attractiveness and comfort of the I-MOVE were rated highest (‘like very much’) by majority of the children. While ease of restraint use was the feature rated highest by parents follow by the attractiveness and safe movement of the system. Parents were also somewhat pleased with the size and comfort of the I-MOVE system.

### Utility of I-MOVE

**Fig. 4:**
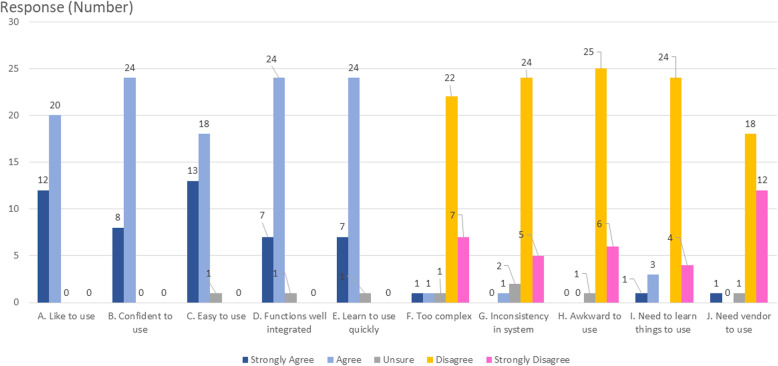
Rating of usability of system by HCPs

Results of the HCP’s group evaluation on utility of the system of I-move is summarized in Fig. 4. All HCPs opined that they would confidently use such a system. Most (96.9%) opined that the system is well integrated and easy to use. Positive response is congruent with vast majority disagreeing that the system was awkward, inconsistent, complex, inability to operate.

### Situational use of I-MOVE

Use of IMOVE in within hospital transport of children is endorsed by HCPs (*n* = 32, 100%) and parents (*n* = 29, 96.7%); induction of anaesthesia for neurotypical children by HCPs (*n* = 32, 100%) and parents (*n* = 28, 93.3%). In addition, use of IMOVE is indicated by children for intra-hospital transfer (*n* = 22, 95.7%), induction of sleep (*n* = 17, 73.9%), dental check-up (*n* = 19, 82.6%) and dental check-up (*n* = 19, 82.6%).

### Use of restraint on children

Twenty-four parents agree to using restraint as long as it is needed for care and treatment. Ten parents agreed as long as it is necessary for their child’s safety. Five parents agreed as long as they feel that their child is not physically hurt, 2 parents agreed if they feel their child is not upset while 1 parent is agreeable if he/she was not upset by the restraint. One parent did not agree for restraint use under any circumstance; the same parent however opined that he did not object to the use of the ‘restraint module’ of I-MOVE as he felt that the restraint module provides safe securing of the child rather than restraining a child.

Free response on likes and dislikes of parents are summarized in Tables 2 and 3 with supporting quotations. Quotations incorporated four subthemes of likes that were common in both parent and children group, these were related to attractiveness, functionality, safety, comfort of use of I-MOVE. In addition, ease of use (*n* = 2) and innovative design (*n* = 2) was identified in the parent group, while 3 children responded that they are happy to be in it. Subthemes relating to dislikes are presented in Table 3.

**Table 2:** Summary of what parents and children like about the I-MOVE system

**What Parents like** 1. **Attractiveness (*n*** **= 14).** Design, shape, colours are appealing and attractive and sporty (for boys) .’ The device resembles amusement park ride, rollercoaster, Virtual Reality chair, arcade (game) chair, office chair’. ‘chair’ design is familiar, children can relate to it easily and has less anxiety and hence not intimated by the device’. ‘Looks like children are going on a ride, makes it easier to get them on it’. ‘The relationship associated with fun in using the chair makes its use less intimidating to children’. 2. **Functionality (*n*** **= 14).** The multifunction and flexibility in usage related to the multi-positional features, its automated design and foot rest. The chair design and restraint ensure safe movement. 2 parents cited ease of use. Useful in ‘movement between different floors during hospital transfer’, ‘holding children in dental chair’ ‘transfer to the OT table and movement in the perioperative period’, making ‘handling of child smoother’. ‘Less chance of injury during transfer’, ‘can replace wheelchair’. 3. **Safety (*n*** **= 9).** The structure and restraint feature. The device design ensures ‘safety precaution and is able to control child with safety feeling’. ‘Over the head chest harness (resembling a seat on roller coaster rides) is a good safety feature for holding children’s head safely during moving after sedation. ‘Looks sturdy for heavy kids. ‘very helpful for ASD patients towards mobility safety measures and concerns.’ 4. **Comfort (*n*** **= 4).**Comfortable. ‘does not look cold or intimidating’. Its ‘bright colours also comforting to the child to sit on it’. 5. **Innovative and creative (*n*** **= 2).**	**What children like** 1. **Attractiveness (*n*** **= 15).** Chair and the amusement park restraint design are described as cool/nice/ high tech/ interesting/ /colourful. ‘The restraint/handle and colour are cool’, ‘like a massage chair or a roller coaster’. ‘it is full of colours, I like it but don’t know why, I want to use the chair’. ‘chair is cool and makes me feel like it would not be a serious operation’. 2. **Functionality (*n*** **= 9).** The ability to move and transform into various shapes and forms for difference uses and ability to hold children with remote control. ‘I like the moving thing about it and that it can move with remote control and can sleep on chair. ‘the chair is versatile and solving many problem’. ‘If child is afraid and wants to run away, seatbelt can hold them’. ‘I like to go to sleep for operation in this chair, I like to sit on it, go round and round in it’. 3. **Safety and security (*n*** **= 4).** ‘Especially important for a child who is unsure what is going to happen’. ‘It is safer to carry child’. 4. **Comfortable** (*n* = 8). ‘comfortable’, ‘Like the speed’, ‘feels happy and like to sit’.

**Table 3:** Summary of what parents and children dislike about the I-MOVE system

**What parents did not like** 1. **None (*n*** **= 5).** 2. **Restrain (*n*** **= 9).** (a) mechanism of the seat belt buckle (*n* = 4) (b) design of overhead chest restraint (*n* = 3). Inadequate restraint (*n* = 2). Input include ‘seat belt not easy to ‘lock and unlock’, ‘place the seat belt buckle at the back so that the child could not unbuckle themselves’, use a ‘4 points seat-belt’, use ‘auto lock over-head chest restraint’, ‘overhead restraint looks intimidating, may get in the way of tall child’. Add ‘more restraints, such as seat belt around the leg. 3. **Colour** (*n* = 6). The current colours (black and red) may not appeal to all children. Input include ‘red colour may appear dangerous and intimidating’. ‘colours may not be appealing for girls.’ use 'calming' colours (like blue), dim shades of colour or more cheerful and happy colours’. 4. **Motor Sound (*n*** **= 4).** 5. **Comfort (*n*** **= 3) relating to the seat cushion.** ‘The head pillow was at a fixed position and could not be removed for more comfort’. ‘Not sure if the pillow is comfortable” 6. **Others (*n*** **= 2).** ‘Device appears too attractive that the child may think it is a computer game chair and is expecting to have some sort of game play’. ‘The bucket seat appears to be like a race car, too fast too furious’.	**What children did not like** 1. **None (*n*** **= 6)** 2. **Seat belt restraint (*n*** **= 6).** Concern include hard to apply, uncomfortable and tight for the bigger child^a^. 3. **Colour of the chair seat** (*n* = 2). ‘Red is too bright’. ‘Suggest black’, ‘Colour is a bit fierce’ 4. **Motor Sound (*n*** **= 2).** 5. **Size** (*n* = 3). ‘The device is big and the head pillow is too high (9, 11 years old children) 6. **Movement (*n*** **= 2).** Concern that children may find it ‘scary’ when chair moves up for those fearful of height’ and ‘if they think that no one is controlling the chair.

^a^Despite the seat belt being adjustable, children find it hard to do so

## Discussion

The design of I-MOVE focuses on both functionality and external appeal; Essential functionality includes ability to restraint and to assist movement and transfer of children through enabling multi-position transformation of the mobility device. The aim is to improve patient safety, increase efficiency and reduce perioperative staff injury during patient transfer to the operating room, induction of anaesthesia and transfer to the operating table. The external appeal of the mobility device aims to promote positive child-machine interaction and enhance patient experience. Here, the seat module of I-MOVE bears likeness to various forms of a chair (racing chair in sitting configuration, recliner massage chair when supine) and the restraint modules resembles those found in airplanes and amusement rides. By integrating elements of familiarity and fun with functionality, we hope to achieve reduction of stress and anxiety and greater acceptance of the mobility device by children.

In recent years, greater understanding and awareness of ASD had led to efforts to optimize management of ASD children in the hospital environment for a better patient experience. At the same time, there has been a paradigm shift towards evidence-based practice in patient handing equipment focusing on ergonomics [17]. The act of transferring patients on and off OR beds has also been identified by the Association of periOperative Registered Nurses Workplace Safety Task Force as a high-risk task for healthcare staff musculoskeletal injury in the perioperative area [11]. Standards relating to safe patient handling and injury prevention now recommend the use of engineering control to perform lifting, transferring and repositioning of patients and eliminating manual lifting of patients when the load exceeds 35 pounds [12].

Anxiety and disruptive behaviour requiring sedation for children with ASD are common in the preoperative period, reported sedation rates in studies ranged from 76 to 87.5% [18–20]. In our hospital, sedation is administered to the patient in the OT waiting area if deemed necessary by the attending anaesthesiologists. After which, the patient waits on a trolley before transfer to the OR. Children who do not require sedation either walk or travel on a trolley, wheelchair or a pull cart to the OR. Parental presence in the OR at induction of anaesthesia is the standard practice. For children who refuse to sit on the operating table, they are placed on parents’ lap during inhalation induction, then lifted and carried onto the operating table by healthcare staff after being anaesthetised. When child becomes anxious and non-compliant, these movements may become difficult, require manpower to hold and restraint, and may put patient at risk of injury during fight or flight. Manual transfer of child from parent’s lap to operating table may also predispose a child to fall risk especially when child is older and heavier.

The utility of the system is well received with vast majority of HCPs (> 95%) agreeing that the system is well integrated, easy to learn and use. Vast majority of parents and HCPs opine favourably for the use of I-MOVE system concept to improve the management of perioperative movement (90.0% parents, 100% HCPs) and to enable safe perioperative movement of children (86.7% parents, 96.9% HCPs) in the situation when children becomes uncooperative. Vast majority of HCPs with experience in the perioperative care of ASD children and parents with ASD children would recommend frequent use of such a system on combative ASD children in clinical setting as well as  recommending the system to others (HCPs and parents). We have recruited perioperative HCPs and parents in the evaluation of the concept mobility device for two reasons. HCPs are clinical expert in management of children with ASD in the perioperative process and gives valuable practical input while parents are content experts in the care of children with ASD and the best resource for developing individualized strategies for a successful experience [8]. The opinion between HCPs and parents on the functionality of the system is not dissimilar, though parental ratings are more conservative. Comfort of anaesthesia and ease of induction of anaesthesia were two domains that were rated the lowest by parents (76.7% agree or strongly agree) and are areas to improve and explore in future.

Parents and children are found to share similar reasons for their likes and dislikes for the mobility device, namely attractiveness, fun and functionality. Attractiveness, comfort and the size of the system were features rated most appealing to children while parents were most appealed by the ease of restraint use. Most children did not find the restraint module intimidating as the design inspires fun (amusement park) and gives the perception of protective stabilization rather than a forceful lock-down. Most parents are also accepting to the use of restraint if it is required for treatment and if applied in a secure and safe manner. Children’s endorsement for use of I-MOVE to ‘go to sleep’ for surgery is lower than its use as dental chair, it is not known if the notion of ‘surgery’ had negatively impacted the children’s opinion, this can be evaluated in future.

There are inherent limitations in this study. Firstly, this is a proof of concept, the evaluation is not based on bed-side use. Active participation from the children in I-MOVE system was conducted in a non-clinical environment. Secondly, the ASD cohort in our study are cognitively abled while general population of children with ASD are known to have intellectual and learning disabilities [21]. Severe ASD patients are often the group that require physical restraint [22] and is the target population of interest. Our findings may not be generalized to those with severe behaviour problem or those who develop aggression during the perioperative period at this juncture. We have included neurotypical children because deployment of system in practice will be extended to all children. Lastly, the survey used to evaluate the functionality of the mobility system in this study is designed by the investigating team and not a validated tool. However, we have however evaluated HCP’s opinions on utility of the mobility system based on System Usability Scale (SUS).

This is one of the first studies that report on perioperative movement of children with ASD. Currently there is no standardized strategies or guidelines for safe transportation targeted exclusively for the perioperative period. This proof-of-concept work represents a multidisciplinary collaborative approach between HCPs, engineers and parents to create an approach to improve the perioperative journey. This study also identifies deficiencies for improvements, the next step is to build on this proof-of-concept and refine the working prototype in a collaborative model between inter-disciplinary healthcare professionals (physicians, nurses, allied health professionals, child psychology), patient experience and local healthcare organizational transformation specialist to innovate new model of hospital transfer for children. Indeed, establishing such a interdisciplinary perioperative team to implement a ‘formal systemized Safe Patient Handling Movement program’ is recommended by Association of periOperative Registered Nurses [11, 13]. Early introduction of I-MOVE early such as during surgery consult or integration of I-MOVE into a social story can help familiarize the children with the concept of I-MOVE for hospital mobility. It may be worthwhile to explore the use of I-MOVE for intra-hospital transfer outside the OT environment for children with ASD to gather clinical use experience.

## Conclusion

A concept mobility device that integrates appeal for child-machine interaction through context of familiarity and fun with functionality of restraint and multi-positional transformation, was opined by vast majority of HCPs and parents of children with ASD to promote safe movement and induction of anaesthesia in anxious children with ASD. The attractiveness and comfort of use of such a mobility system makes it a potentially useful modality for intra-hospital transfer for children with ASD.

## Data Availability

The datasets used and/or analysed during the current study are available from the corresponding author on reasonable request. All data generated or analysed during this study are included in this published article.
